# Effects of Acupuncture on Heart Rate Variability in Beagles; Preliminary Results

**DOI:** 10.1155/2013/419212

**Published:** 2013-03-27

**Authors:** Huan Wang, Gerhard Litscher, Xian Shi, Yue Bo Jiang, Lu Wang

**Affiliations:** ^1^Department of Acupuncture, People's Liberation Army General Hospital, Beijing 100853, China; ^2^Stronach Research Unit for Complementary and Integrative Laser Medicine, Research Unit of Biomedical Engineering in Anesthesia and Intensive Care Medicine and TCM Research Center Graz, Medical University of Graz, 8036 Graz, Austria

## Abstract

Evidence-based animal experimental research concerning the effects of acupuncture on autonomic function was performed by two research teams from China and Austria. This study describes measurements in beagles. Heart rate variability (HRV) recordings were performed under stable conditions in Beijing, China, and the data analysis and interpretation were completed in Graz, Austria. The electrocardiograms were recorded during bilateral body acupuncture (PC6, Neiguan). Power of the low frequency (LF), high frequency (HF), and the ratio (LF/HF) changed significantly during acupuncture stimulation in beagles after injection of atropine and **β**-blocker. However, there was no significant change in HF power after needling the Neiguan acupoint when a cervical vagotomy has been performed. Our findings show that acupuncture can mediate the HRV even after pharmaceutical blocking of autonomic function. Acupuncture effects on HRV should rely not only on autonomic nervous system but on complete central nervous system.

## 1. Introduction

Acupuncture is being recognized as an effective treatment for various autonomic disorders; however, most of the mechanisms of this therapeutic method still remain unclear. The results obtained using good experimental designs are well documented and are important for general acceptance of this traditional Chinese medical treatment in the Eastern and Western worlds.

This study represents different acupuncture measurements performed on beagles. The main goal was to investigate the effects of acupuncture stimulation on heart rate variability (HRV) in dogs under stable conditions. The data were recorded in Beijing, China, and the data analysis and interpretation were completed in Graz, Austria. 

## 2. Materials and Methods

### 2.1. Animals

Three beagles (weight 27.5 kg, 25 kg, and 30 kg) were investigated. Each dog was housed in a well-ventilated facility in a single cage and fed twice a day with commercial dry food. The animals were anesthetized with an intraperitoneal injection of 2.5% pentobarbital sodium (1.0 g/kg). Experiments were conducted in accordance with the Guide for Care and Use of Laboratory Animals issued by the National Institutes of Health, and the procedures were approved by the Institutional Animal Care Committee.

The exact anatomical features, especially the centers in the beagles' brain responsible for the modulation of HRV, are described in detail in special books [1].

### 2.2. Electrocardiographic Monitoring

The electrocardiogram (ECG) was monitored using a Zymed 1410-type 3-lead dynamic ECG system (La Honda, CA, USA). After fast Fourier transformation (FFT) into heart rate spectra, low frequency (LF; 0.04–0.15 Hz) was used to represent the usual regulating region by sympathetic and vagus nerve activity, and high frequency (HF; 0.15–0.40 Hz) represented the vagus nerve activity. In addition, the LF/HF ratio was calculated. In order to minimize the number of animals, we analyzed altogether nine measurement phases in three dogs. The measurements were repeated two times in all dogs.

### 2.3. Acupuncture Stimulation, Drugs, and Procedure

**Figure 1:**
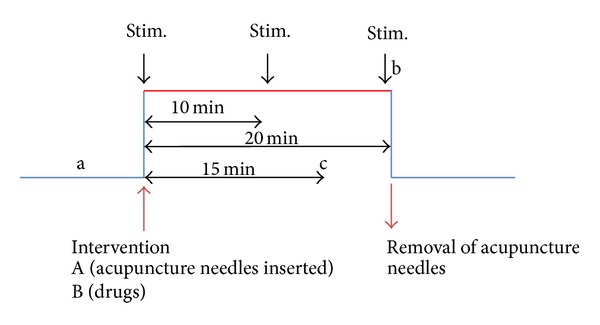
Measurement procedure of the study.

Once the data of the ECG monitoring had reached a steady state, acupuncture stimulation was performed. For manual acupuncture stimulation, sterile single-use needles (length: 30 mm, diameter: 0.3 mm; Huan Qiu, Suzhou, China) were inserted perpendicularly to the skin to a depth of approximately 15 mm at the Neiguan acupoint (PC6) on both sides. The needles were stimulated clockwise and counterclockwise for 15 sec each, with two rotations per second, resulting in 30 rotations per stimulation. The stimulation was performed immediately after inserting the needle, 10 min later, and before removing the needle, always by the same expert. After 20 min, the needles were removed. All points were identified by anatomical marks based on descriptions in textbooks [2]. Briefly, PC6 is located proximal to the accessory carpal pad of the forelimb between the flexor carpi radialis and palmaris longus ligaments. It is one of the most prominent acupuncture points. The following measurement periods were analyzed: one before intervention (a) (acupuncture (A) or drugs (B)), one after 30 sec at the end of three acupuncture stimulations (b), and 15 min after injection of drugs (c), respectively (Figure 1). As already mentioned, the dogs were also investigated under the influence of propanolol (0.2 mg/kg body weight, partial *β*-adrenergic blockade) and atropine (0.1 mg/kg body weight, partial vagal blockade).

### 2.4. Statistical Analysis

The data were analyzed using one-way repeated measures analysis of variance (ANOVA) (SigmaPlot 11.0, Systat Software, Chicago, USA). Post hoc analysis was performed using Tukey and Holm-Sidak tests. The level of significance was defined as *P* < 0.05.

## 3. Results

Tables 1–3 demonstrate the total results from the parameters (mean values ± standard deviation) of HRV in the nine measurements after drug application, after acupuncture, and after vagotomy.

After injection of atropine and propranolol dinitrate, HRV shows the changes of LF, HF, and LF/HF. Both drugs induced a significant decrease in LF (*P* < 0.05). The injection of propranolol led to a significant (*P* < 0.01) increase in HF which contributed to the significant (*P* < 0.01) decrease in LF/HF. The atropine decreased the HF significantly (*P* < 0.01), which led to a significant (*P* < 0.01) increase in LF/HF (see Table 1).

The effects of acupuncture on HRV after blocking the autonomic function by atropine and propranolol dinitrate can be seen in Table 2. After giving atropine and propranolol dinitrate, the LF and HF of the animals decreased significantly compared with the control measurement (*P* < 0.05), and LF/HF was increased significantly (*P* < 0.05). After the acupuncture treatment, both LF and HF increased, but only HF increased significantly (*P* < 0.01), which led to significant (*P* < 0.01) changes in LF/HF (see Table 2).

Table 3 summarizes the effect of acupuncture on HRV after cervical vagotomy. Continuous ECG monitoring showed substantial and significant decreases in both the LF and HF band (*P* < 0.01) and significant increases in LF/HF ratio (*P* < 0.01). After acupuncture stimulation, both LF/HF and LF increased significantly (*P* < 0.01, *P* < 0.01, resp.), but there was no significant change in HF. 

## 4. Discussion

There are several human and animal HRV investigations concerning the mediation of this physiologic parameter. On the one hand the HRV can be blocked through vagotomy of the Nn vagi [3] but also atropine can depress HRV depending on the dosage [4, 5] which is also demonstrated in this study. On the other hand a *β*-receptor blockade has no or only minimal effect on HRV [4, 5]. Investigations in animal models show that changes in the heart rate after stimulation of the sympathicus are independent of the cardiac cycle. In contrary after vagal stimulation there is a manifestation in relationship to the heart cycle with an irregular, discontinuous function of the vagal stimulus frequency. This could be found in the ECG as alterations between the different activities of the heart chambers [6]. These results show clearly that the HRV is mainly influenced via parasympathetic activity; only the low frequency parts will be mediated from sympathetic and parasympathetic nervous system simultaneously [5, 7]. These activities are mainly based on cardio circulatory centers in the medulla [8, 9].

There are several animal experimental studies in the field of HRV in dogs available in the scientific literature [10–15]. The HRV reduction in our study was mainly due to the reduced vagus nerve activity and increased sympathetic nerve activity. Vagus nerve's function is to protect the heart [16]. Our report supports the idea that vagus nerve activation can increase the LF and reduce the HF and HRV, which coincides with previous studies. The present experiment shows clearly that needling the acupuncture point Neiguan can significantly improve the HRV of beagles with low HRV level induced by atropine. So needling Neiguan acupuncture point plays a role of drug intervention effect resistance which can inhibit the sympathetic nervous system and activate vagus nerve activity at the same time. It is interesting that needling PC6 can also regulate sympathetic nervous system when the sympathetic nerve activity was inhibited by *β*-blocker. HRV decreased significantly when both sympathetic nervous system and vagus nerve activity were blocked, and the effects of acupuncture on HRV were inhibited significantly, suggesting that complete autonomic system is necessary in acupuncture treatment to balance the function of sympathetic nervous system and vagus nerve activity. Considering the insignificant regulation after needling Neiguan acupoint and blocking both ways of sympathetic nervous system and vagus nerve, we deduce that acupuncture still works through the humoral coordination. Acupuncture mechanism seems to work in two directions. It can help the organism return to its own balance. 

The data mainly suggest that acupuncture can only mediate sympathetic nerve function after vagotomy.

There are some limitations within this pilot study. The number of animals was small; however as already mentioned we wanted to minimize the number of animals and therefore we analyzed altogether nine measurement phases. In addition there was no control group with a control nonacupuncture point.

Of course, HRV can be calculated not only in the frequency domain, but also in the time domain. However, with this kind of analysis it is not possible to differentiate between the single frequency components, and therefore it was not used in our present study [8, 17].

As already mentioned, Neiguan (PC6) is a prominent and classic acupuncture point in traditional Chinese medicine. It is considered to be effective in the treatment of cardiovascular disorders. In a recently published review article [18], the authors focused on the neurophysiological bases of the effects of PC6 stimulation on cardiovascular mechanisms. They stated that experimental studies have shown that the hypothalamic rostral ventrolateral medulla, arcuate nucleus, and ventrolateral periaqueductal gray are involved in acupuncture-induced attenuation of sympathoexcitatory cardiovascular reflex responses [18]. This long-loop pathway also appears to contribute to the long-lasting, acupuncture-mediated attenuation of sympathetic premotor outflow and excitatory cardiovascular reflex responses [18]. Thus, the authors conclude that acupuncture of PC6 modulates the activity in the cardiovascular system, an effect that may be attributed to an attenuation of sympathoexcitatory cardiovascular reflex responses [18].

After bilateral cervical vagotomy needling and stimulation of the acupuncture point Neiguan can still evoke HRV changes. We deduce that acupuncture can also work through humoral coordination. This could be an interesting topic for future investigations.

## 5. Conclusions

The following conclusions can be drawn from the results of this transcontinental experimental animal acupuncture study.

- Both propranolol and atropine can affect the HRV of beagles significantly.
- Acupuncture can mediate the HRV even after pharmaceutical blocking of autonomic function.
- Acupuncture effects on HRV should rely not only on autonomic nervous system but on complete central nervous system.

## Figures and Tables

**Table 1:** Drug effect on HRV.

Phase	LF (bpm^2^)	HF (bpm^2^)	LF/HF
Control	37.27 ± 16.20	45.02 ± 25.32	0.91 ± 0.32
Propranolol	19.25 ± 8.24*	66.83 ± 26.26**	0.68 ± 0.35**
Atropine	19.88 ± 20.10*	5.03 ± 6.44**	6.89 ± 3.19**

*P < 0.05; **P < 0.01 (LF: low frequency band; HF: high frequency band; LF/HF: ratio; bpm: beats per minute).

**Table 2:** Effect of acupuncture on HRV after blocking the autonomic function.

Phase	LF (bpm^2^)	HF (bpm^2^)	LF/HF
Control	37.27 ± 16.20	45.02 ± 25.32	0.91 ± 0.32
Atropine/propranolol	13.97 ± 5.72*	14.15 ± 9.30**	1.83 ± 1.77**
Acupuncture	14.44 ± 5.75*	22.97 ± 17.45**	1.57 ± 1.05**

*P < 0.05; **P < 0.01 (LF: low frequency band; HF: high frequency band; LF/HF: ratio; bpm: beats per minute).

**Table 3:** Effect of acupuncture on HRV after cervical vagotomy.

Group name	LF (bpm^2^)	HF (bpm^2^)	LF/HF
Control	37.27 ± 16.20	45.02 ± 25.32	0.91 ± 0.32
Cervical vagotomy	1.60 ± 0.04**	0.28 ± 0.03**	5.63 ± 0.22**
Acupuncture	1.86 ± 0.86*	0.28 ± 0.16	6.38 ± 1.46**

*P < 0.05; **P < 0.01 (LF: low frequency band; HF: high frequency band; LF/HF: ratio; bpm: beats per minute).
